# A New CD10 Antibody Inhibits the Growth of Malignant Mesothelioma

**DOI:** 10.1089/mab.2020.0033

**Published:** 2021-02-24

**Authors:** Natsuko Mizutani, Masaaki Abe, Kazunori Kajino, Shuji Matsuoka

**Affiliations:** ^1^ Department of Medical Technology, Faculty Health Sciences, Kyorin University, Tokyo, Japan.; ^2^ Department of Immunological Diagnosis, Graduate School of Medicine, Juntendo University, Tokyo, Japan.; ^3^ Department of Pathology, Oncology and School of Medicine, Juntendo University, Tokyo, Japan.; ^4^ Department of Human Pathology, School of Medicine, Juntendo University, Tokyo, Japan.; ^5^ Division of Cancer Immunotherapy, Exploratory Oncology Research and Clinical Trial Center, National Cancer Center, Kashiwa, Japan.

**Keywords:** monoclonal antibody, malignant mesothelioma, CD10, JMAM-1

## Abstract

Malignant mesotheliomas (MMs) are aggressive therapy-resistant tumors that generally have a poor prognosis. We previously reported the establishment of four new monoclonal antibodies (mAbs) for the diagnosis and treatment of MM. In this report, we characterized one of these antibodies, JMAM-1. The molecules whose antibodies were calibrated were picked up, transfected assuming CD10, and elucidated by fluorescence activated cell sorter. Survival experiments were performed using tumor-bearing mice model. JMAM-1 mAb was found to bind with CD10 antigen. The Kaplan–Meier survival curve showed a small but prolonged survival effect. JMAM-1 mAb-treated MSTO-211H cells showed increased cell cycle arrest involved by cyclin-dependent-kinase. JMAM-1 antibody has cytostatic effect and may be a candidate for the treatment of MM. Among mesothelioma, CD10-positive cases have been reported to have a poorer prognosis than negative cases, which can be used as a tool for diagnosis.

## Introduction

Malignant mesothelioma (MM) is an uncommon but aggressive tumor with a very poor prognosis. Despite improvements in surgical management, chemotherapy, and radiotherapy, its prognosis remains poor, with a median survival of <2 years.^(1–7)^For clinical diagnosis, patients with the sarcomatoid subtype have the poorest prognosis with a remarkably short survival.^(8)^ Even among patients with epithelioid mesothelioma, survival outcomes are variable. Therefore, further prognostic factors are necessary to optimize treatment options and to better stratify patients in clinical trials.^(9,10)^

CD10 (neutral endopeptidase), a zinc-dependent metalloproteinase, is expressed in various normal tissues and is capable of efficiently degrading various peptides and cytokines.^(11–14)^ CD10 is also expressed in malignant tumors and has been identified as a predictor of tumor biological aggressiveness through extracellular enzymatic degradation and intracellular signaling crosstalk.^(15–25)^ CD10 is expressed in MM,^(26)^ and patients present with a poorer prognosis than negative cases.

Recently, CD10 has been demonstrated to be a novel marker of cisplatin resistance and cancer stem cells using cell lines from other solid malignancies.^(27)^ In addition, CD10 has been reported to cleave and activate a peptidic prodrug of doxorubicin,^(28,29)^ and recent clinical trials suggest that chemotherapy with doxorubicin improves quality of life with an acceptable level of toxicity.^(30,31)^ Therefore, CD10 is a potential marker for investigating chemotherapy sensitivity or resistance in patients with MM.^(26)^ These results indicate that CD10 is closely related with tumorigenicity and self-renewal ability. Furthermore, tumoral CD10 expression correlates with aggressive histological types and higher mitotic activity, and it is an independent prognostic factor for patients with MM.

In the first report, we determined the establishment of four antibodies against MM. However, at that time, the antigen molecules of each antibody had not been identified.^(1)^ Herein we report the identification of the antigen molecule and other studies on the JMAM-1 antibody, which had the highest cell growth inhibitory effect, among the four antibodies.

## Materials and Methods

### Ethics approval and consent to participate

Animal experiments were conducted following protocols approved by the Animal Care Committee of the Juntendo University of Medicine. The Ethics Review Committee for Animal Experimentation at the Juntendo University Faculty of Medicine approved all animal experiments (Project Number 260105).

### Animals

Female BALB/c nu/nu mice of 4 weeks of age were obtained from SLC (Hamamatsu, Japan) and housed in a specific pathogen-free facility in microisolator cages. The Animal Care and Use Committee of Juntendo University approved all animal experiments.

### Cell lines

NCI-H226 and MSTO-211H mesothelioma cell lines and Huh-7 hepatoma cell lines were purchased from American Type Culture Collection. Cells were cultured in RPMI-1640 supplemented with 10% fetal bovine serum (FBS; Thermo Fisher) in standard conditions (5% CO_2_ at 37°C). Cells undergoing exponential proliferation were used for all experiments.

### Reagents and antibodies

Mouse anti-human leukocyte antigen (HLA) class I (HLA-A, -B, and -C) monoclonal antibody (mAb; clone: W6/32) was purchased from BioLegend (San Diego, CA). Alexa Fluor 488-conjugated goat anti-mouse IgG was purchased from Invitrogen (CA). Mouse IgG was purchased from Abcam (Cambridge, United Kingdom). Anti-CD26 mAb (clone 1F7) and ERC-mesothelin were established in our laboratory.^(32,33)^ Anti-CD10 mAb (clone 56C6) was purchased from LSI Medience. EnVision™+DualLink (DAKO) and 3,3′-diamin-obenzidine (Dojindo Laboratories) were used as the chromogens. Alexa 488 conjugate was purchased from Thermo Fisher.

### Plasmid

RG223013 (Qiagen, Stockholm, Sweden) and FuGENE^®^ 6 reagent (Promega, Japan) were used.

### Transfection of chimeric construct and establishment of stable transfected cell lines

Twenty-four hours before transfection, 2 × 10^5^/mL Huh-7 cells were seeded in a 60-mm plate. The RG223013 construct was prepared using Plasmid Maxiprep (Qiagen), and then, the Huh-7 cells were transfected with FuGENE 6 reagent (Promega) according to the manufacturer's instructions. At 8 hours, the culture medium was replaced with RPMI-1640 medium containing 10% FBS. After 48 hours, the supernatant of the transfected cells were confirmed by flow cytometry analysis using a BD LSRFortessa cell analyzer (BD Biosciences). The cells were collected and analyzed for transient expression of CD10 mAb through western blot analysis.

### Western blot analysis

MSTO-211H, Huh-7 cells, and transfected Huh-7 cells (3 × 10^6^ cells/well) incubated, with or without rfhSP-D (20 μg/mL), in a serum-free Roswell Park Memorial Institute (RPMI) medium for 12 and 24 hours. The cells were lysed in a lysis buffer (50 mM Tris-HCL pH 7.5, 150 mM NaCl, 1% Triton X, 1 mM sodium orthovanadate, 10 mM β-glycerophosphate, 2 mM ethylenediaminetetraacetic acid (EDTA), and 10 mM sodium pyrophosphate) and analyzed by western blotting. Lysate proteins (30 μg) were separated by 15% sodium dodecyl sulfate - polyacrylamide gel electrophoresis (SDS-PAGE) and electrophoretically transferred onto poly vinylidene di fluoride (PVDF) membranes (Pall Corporation, NY, USA). Protein samples were separated by SDS-PAGE and transferred to a PVDF membrane. After treatment with Pierce Fast Blocking Buffer (Pierce Biotechnology, Inc., Tokyo, Japan), the membranes were incubated with a buffer containing JMAM-1 mAb (1 μg/mL), followed by a horseradish peroxidase-conjugated secondary antibody. The membrane was treated with an enhanced chemiluminescent reagent (Amersham, Tokyo, Japan), and the reactive protein bands were visualized using a Fujifilm image analyzer.

### Cell viability assay

MSTO-211H cells were seeded in triplicate in 6-well plates at a density of 3 × 10^5^ cells/well. After 24 hours, JMAM-1 mAb was added to each well at a concentration of 5 μg/mL. The percentage of viable cells after treatment was calculated assuming 100% as the number of untreated cells. We counted the number of viable cells 24, 48, and 72 hours after antibody administration. Cell viability was then determined by a dose–response curve using GraphPad Prism 7 Software.

### Statistical analysis

All experiments were conducted in triplicates, and the data are expressed as mean ± standard deviation (SD). The resulting mean values were <10% SD. Statistical analyses were performed using the SPSS 14.0 software (SPSS, Inc., NY). The data sets were compared by Student's *t*-tests, and *p* < 0.05 was considered statistically significant.

## Results

### JMAM-1 mAb binds with CD10 antigen

**FIG. 1.**
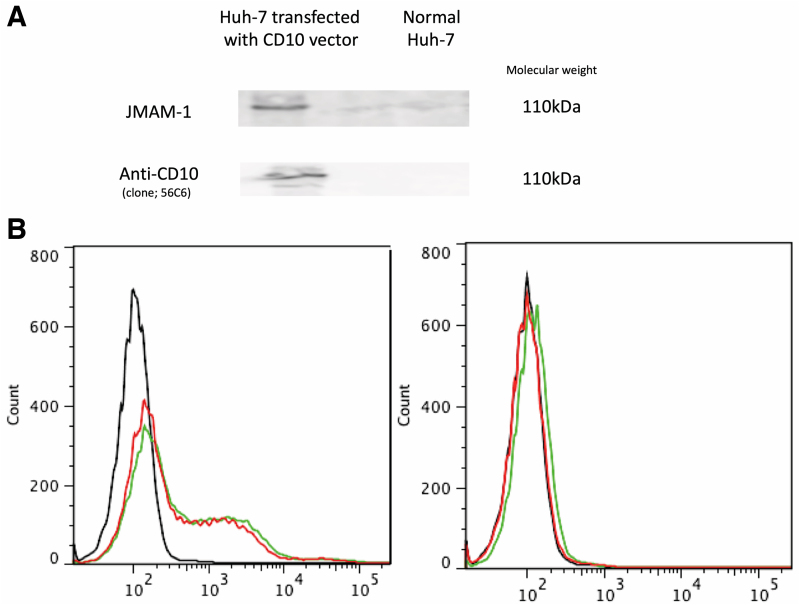
JMAM-1 mAb recognized CD10 transfectant. Right side indicates normal Huh-7 cells, and left side indicates CD10 vector-transfected Huh-7 cells **(A, B)**. **(A)** Western blotting indicated anti-CD10 expression by transfected Huh-7 cells. **(B)** FACS proved that JMAM-1 mAb recognized anti-CD10. Black line, control mouse IgG. Red line, anti-CD10 mAb. Green line, JMAM-1 mAb. FACS, fluorescence activated cell sorter; mAb, monoclonal antibody.

When a comprehensive analysis of the antigen molecule was requested for JMAM-1 mAb, the possibility of CD10 was suggested, and it was necessary to prove it by transfecting nonexpressing cells. When examined by fluorescence activated cell sorter (FACS), Huh-7 cells usually do not bind to the JMAM-1 antibody. When FACS was subsequently performed by introducing the CD10 plasmid into the cells, binding to the JMAM-1 antibody was observed. Therefore, it was suggested that the JMAM-1 mAb was anti-CD10 mAb (Fig. 1). Western blot analysis indicated same molecular weight.

### JMAM-1 mAb-treated MSTO-211H cells showed increased cell cycle arrest

**FIG. 2.**
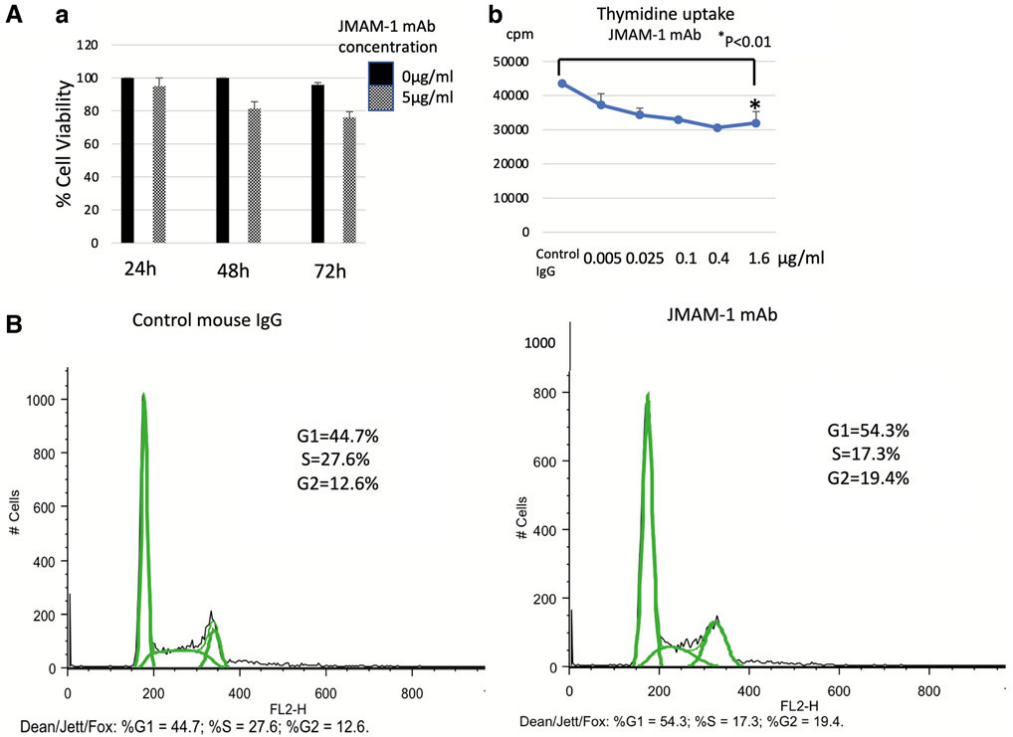
JMAM-1 mAb-treated MSTO-211H cells showed increased cell cycle arrest. **(A)** (a) Effect of JMAM-1 mAb on cell viability. MSTO-211H cell viability was analyzed by JMAM-1 mAb treatment (24–72 hours). (b) Effect of JMAM-1 mAb on thymidine uptake assay. MSTO-211H cells were incubated with different doses of JMAM-1 mAb (0–1.6 μg/mL) for 72 hours in a CO2 incubator. **(B)** JMAM-1 mAb-treated MSTO-211H cells showed increased cell cycle arrest. MSTO-211H cell was incubated in the presence of JMAM1 (5 μg/mL) or in culture medium alone (control) for 48 hours and was subjected to FACS cell cycle analysis. The total population of cells was divided into the G1, S, and G2 phases and analyzed. Control mouse IgG (%G1 = 44.7, %S = 27.6, %G2 = 12.6). JMAM-1 mAb (%G1 = 54.3, %S = 17.3, %G2 = 19.4). Analyzed by Dean-Jett-Fox.

MSTO-211H cells (2 × 10^5^/well in tissue culture plate) starved for 18 hours in RPMI medium, and then treated with JMAM-1 mAb (5 μg/mL) for 48 hours. MSTO-211H cells were incubated in the presence of JMAM-1 (10 μg/mL) or in a culture medium alone (control) for 24 hours and were subjected to FACS cell cycle analysis. The total cell population was divided into G1, S, and G2 phases and subsequently analyzed (Fig. 2).

### Tumor xenograft experiments

**FIG. 3.**
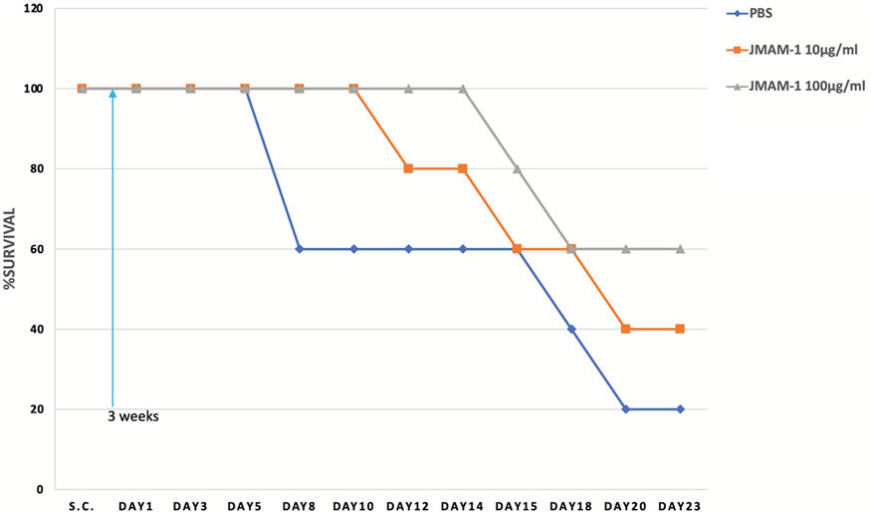
*In vivo* efficacy of JMAM-1 mAb in the NCI-H226 CDX model BALB/c nude mice were injected S.C. with NCI-H226 cells. After 3 weeks, 10 and 100 μg/mL JMAM-1 mAb and control PBS, respectively, were administered intraperitoneally from days 1 to 23, every 2 or 3 days for 11 times. PBS, phosphate-buffered saline.

Female BALB/c nu/nu mouse of 4 weeks of age were obtained from SLC. Thirty BALB/c mouse were injected intraperitoneally with NCI-H226 cells (1 × 10^6^/head) suspended in PBS. Three weeks later, the mice were injected intraperitoneally with JMAM-1 mAb (10 or 100 μg/mL/head) or with control PBS 11 times between days 1 and 23. A slight but prolonged survival effect was observed in the group administered 100 μg/head compared with the group administered 10 μg/head and the PBS group (Fig. 3).

### Comparison between JMAM-1 and other mAbs by XTT assay

**FIG. 4.**
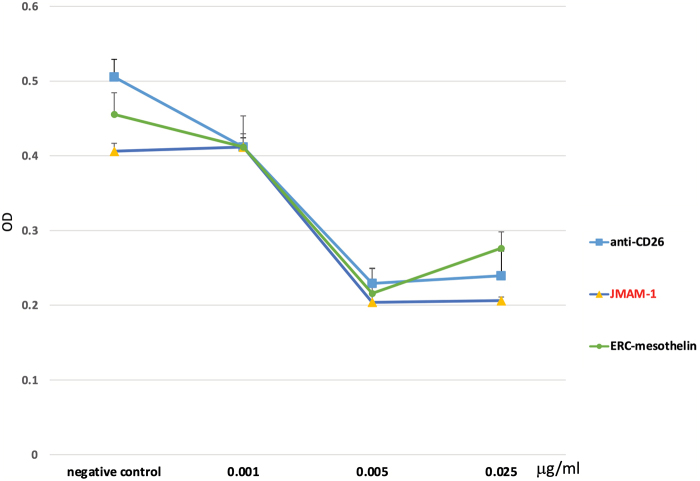
Comparison between JMAM-1 and other mAbs by XTT assay. NCI-H226 cells (3 × 10^3^ cells/well) were seeded in a 96-well plate and XTT assay was performed. JMAM-1 concentrations used for dose–response analyses were 0.001, 0.005, 0.025, 0.1, 0.4, and 2 μM. After 48 hours, ERC-mesothelin and anti-CD26 were prepared using the same concentrations and absorbance was read at 450 nm using an ELISA plate reader (Bio Tek Instruments, Northern Vermont, USA). Data were analyzed using Prism 5 statistical software (*n* = 3; *p* < 0.05) through one-way analysis of variance, followed by Tukey test. JMAM-1 mAb showed almost the same cell growth inhibitory effects as anti-CD26 and mesothelin.

NCI-H226 cells (3 × 10^3^ cells/well) were seeded in a 96-well plate and XTT assay was performed. JMAM-1 concentrations used for dose–response analyses were 0.001, 0.005, 0.025, 0.1, 0.4, and 2 μM. After 48 hours, ERC-mesothelin and anti-CD26 were prepared using the same concentrations and absorbance was read at 450 nm using an ELISA plate reader (BioTek Instruments, Northern Vermont). Data were analyzed using Prism 5 statistical software (*n* = 3; *p* < 0.05) through one-way analysis of variance, followed by Tukey test. JMAM-1 mAb showed almost the same cell growth inhibitory effects as anti-CD26 and mesothelin (Fig. 4).

## Discussion

In our first report, we produced four antibodies (JMAM1, 2, 3, and 4)^(1)^; when one of these antibodies was analyzed, it was possible to identify the molecule was CD10 that recognized. Kadota et al. reported that CD10 expression is positively cases in mesothelioma correlated with higher-grade histological types: CD10 expression was identified in 42% of epithelioid nonpleomorphic tumors, 57% of epithelioid pleomorphic tumors, 79% of biphasic tumors in the sarcomatoid area, and 93% of sarcomatoid tumors. Furthermore, mitotic counts were significantly higher in CD10-positive tumors than in CD10-negative tumors. Moreover, there is an association between tumoral CD10 expression and overall survival in all patients with epithelioid and nonepithelioid mesothelioma.^(6,7)^

For diagnosis of mesothelioma, it is necessary to use the new antibody together with the currently used antibody. However, because it stained both epithelial and sarcomatoid type MM, it seems to be useful for diagnosis. It must be noted that renal cancer was also stained. Thus, for the diagnosis of carcinoma, CD10 mAb is used to establish the origin. Moreover, for MM, it seems that the CD10-positive histological type has a poor prognosis. Thus, CD10 mAb is expected to be used not only for the diagnosis of mesothelioma but also as a prognostic marker for cancer cases.^34^

Currently, we do not know how the epitope of other companies' CD10 antibodies differ. Moreover, identification of epitopes is difficult but will be undertaken if required.

JMAM-1 mAb has an inhibitory effect on cell proliferation, and even Kaplan–Meier estimates showed a life-prolonging effect of JMAM-1 mAb compared with that of control PBS.

However, after repeated therapy, not all patients respond to therapeutic mAbs^(35)^ because clones appear during the course of treatment that do not express the target. Therefore, additional therapeutic options are required to treat such patients. Common therapeutic mAbs against cell surface molecules exert their effects largely through immunological mechanisms, including complement-dependent cytotoxicity (CDC) and antibody-dependent cellular cytotoxicity (ADCC). When we examined whether there are steps that suppress cell growth involving factors other than ADCC and CDC, the cell cycle system was considered.

A cell cycle assay was performed to elucidate cell proliferation inhibition by JMAM-1 mAb, and it was found that fewer cells reached S phase compared with those in the control PBS group, demonstrating inhibition of proliferation. Through G1 to S phase, some cyclin-dependent kinase inhibitory proteins may act in the suppression of cell growth.^(36)^ YAP protein is involved in the proliferation of MPM cells, whereas suppressing CDK7 reduces YAP protein as well as suppresses the infiltration and metastasis of MM.^(37)^ Jinbai reported that CDK7 might be a therapeutic target for MM. CDK7 is the catalytic subunit of the CDK-activating kinase (CAK) complex that phosphorylates SPT5/SUPT5H, SF1/NR5A1, POLR2A, p53/TP53, CDK1, CDK2, CDK4, CDK6, and CDK11B/CDK11. CAK activates the cyclin-associated kinases CDK1, CDK2, CDK4, and CDK6 by threonine phosphorylation, thus regulating cell cycle progression.^(38)^

It may be necessary to examine the relationship between CDK and CD10 in the future.

## Conclusions

JMAM-1 mAb was found to be a CD10 protein. The diagnosis of MM, expression of CD10 correlates with aggressive histological types and is an independent predictor of patient survival. JMAM-1 mAb may also predict the prognosis of MM. Our data show that JMAM-1 mAb inhibits the proliferation of MM cells. This is thus an effective treatment for MM.
